# An Area‐Level Study on Paediatric Burn Injuries and Their Association With Parental Education

**DOI:** 10.1002/puh2.70113

**Published:** 2025-09-02

**Authors:** Gabriela Valladares Solano, Emma Kos, Mattias Wennergren, Ann‐Sofie Frisk Cavefors, Johanna Räntfors, Stefan Nilsson

**Affiliations:** ^1^ Institute of Health and Care Sciences, Sahlgrenska Academy University of Gothenburg Gothenburg Sweden; ^2^ Queen Silvia Children's Hospital, Sahlgrenska University Hospital Gothenburg Sweden; ^3^ General Practice, Family Medicine, School of Public Health and Community Medicine, Institute of Medicine, Sahlgrenska Academy University of Gothenburg Gothenburg Sweden; ^4^ Research, Education, Development and Innovation, Primary Health Care, Region Västra Götaland Gothenburg Sweden; ^5^ Department of Child Health Services Region Västra Götaland Gothenburg Sweden; ^6^ Centre For Person‐Centred Care University of Gothenburg Gothenburg Sweden

**Keywords:** burns, child health services, educational status

## Abstract

**Introduction:**

This study aims to investigate the association between young children receiving hospital treatment for burn injuries and their parents’ education levels.

**Method:**

A retrospective register study collected data using diagnostic codes (T20–T25 and T29) for children seeking emergency medical care for burn injuries between 2018 and 2022. T20–T25 and T29 refer to burn injuries, where each number specifies the affected body region. An additional selection was made using ICD code chapters S and T for overall injury‐related hospital care. The inclusion criteria for this study were children aged 0–6 years who had received emergency or specialised care at Queen Silvia Children's Hospital. The education levels of parents, as well as those of children who received care at the hospital, were grouped and aggregated at the area level. Parental education level data were obtained from Gothenburg's public statistical database to determine education levels in the city's areas. The mean of these two variables was calculated, and a simple linear regression was conducted. Data were aggregated and analysed at the intermediate area level rather than the individual level.

**Results:**

The results showed an association between children's burn injuries and parents’ education levels; however, parental education level and overall injury‐related hospital care were not associated with eachother.

**Conclusions:**

This study demonstrated an association between burn injuries and parental education levels, which needs consideration in clinical practice and future research. Future efforts should explore targeted health literacy and parental support interventions in low‐education areas.

## 1 Introduction/Background

Paediatric burn injuries are most common in the 0–5‐year age group, with an increased risk up to 2 years of age [1, 2, 3]. The most common burn injury in children is scalding, which involves exposure to hot liquids, such as boiling water or tea [1]. Research shows that the socioeconomic status of the child's parents plays a significant role in determining the occurrence of burn injuries [4, 5, 6].

Low education level is a known risk factor for poor health, with individuals possessing lower education levels being at higher risk of health issues compared to those in highly educated groups [7, 8, 9]. Good health correlates with higher education levels, which often lead to improved working conditions, higher wages, more knowledge, better living conditions and improved lifestyle habits [7, 8, 9, 10].

The previous research outside Sweden has suggested that parental education levels correlate with child accidents. Consequently, children with parents who have low education levels may find themselves in more vulnerable situations than those with highly educated parents [8].

In Sweden, all children are entitled to free‐of‐charge healthcare, and most attend regular health check‐ups at the child health service. According to the National Board of Social Affairs and Health and the Swedish child health units, all parents should be educated about child accidents, including burn injuries and their prevention [11]. The Swedish child health service is based on proportionate universalism, which means that all families are offered basic support, but those with greater needs receive additional assistance accordingly [12].

A burn injury can be traumatic for both the child and the family. It incurs multiple costs, making the development and improvement of preventive measures highly beneficial. With a better understanding of risk factors, more effective interventions can be designed in primary care, and improved support for parents can be developed [13, 14].

This study aimed to investigate the association between children being hospitalised for burn injuries and their parents’ education levels.

The study questions were as follows:

- How does parental education level at an intermediate area level correlate with the rate of paediatric burn hospitalisations in Gothenburg?
- What are the differences in paediatric burn injury rates across Gothenburg areas with varying parental education levels?
- To what extent can parental education levels explain the variation in paediatric burn injuries across different regions of Gothenburg?

## 2 Methods

### 2.1 Selection

The inclusion criteria were all children aged 0–6 years who were registered residents of Gothenburg and sought treatment for burn injuries, regardless of burn severity, at Queen Silvia Children's Hospital between 2018 and 2022. The diagnostic groups of interest were those with ICD codes T20–T25 and T29. An additional search, including ICD chapters S and T, was performed. ICD codes T20–T25 refer to anatomical locations of burn injury, whereas T29 is used when multiple body areas are affected by burns. ICD chapters S and T cover a wide range of injuries from external causes [15]. In this study, these codes were used to differentiate children with burn injuries from those with other childhood injuries or trauma.

For parental education levels, ‘pre‐upper secondary programmes’ were classified as low educational attainment, whereas ‘post‐upper secondary programmes’ were classified as high educational attainment for both mothers and fathers at the intermediate area level. Data for the ‘secondary education’ and ‘information missing’ categories were available but not used, as they were considered planned omissions; they did not directly correspond to the study's purpose of investigating the association between parental education levels and burn injuries. To address the research question, the highest and lowest available education levels were deemed sufficient.

The period and geographical selection were carefully considered to obtain a sufficiently large and relevant dataset. The study was limited to Gothenburg, Sweden's second‐largest city, where Queen Silvia Children's Hospital houses one of the country's largest paediatric hospitals and emergency departments. This approach allowed for the inclusion of a larger number of children, making the sample representative of children and parents in the city of Gothenburg.

### 2.2 Data Collection

Data collection was performed in several steps. First, data were collected on children aged 0–6 years who sought treatment for burn injuries at Queen Silvia Children's Hospital between 2018 and 2022. This was done by requesting data based on the previously mentioned diagnostic codes (T20–T25 and T29). To obtain relevant results, given the varying population in each intermediate area, the proportion of children in each area was considered. To enable comparison, if the observed proportion of children who received care for burn injuries was higher than expected, we included the number of children who received care for any type of external injury or trauma (ICD code chapters S and T).

Parental education level data were obtained from Gothenburg's public statistical database to determine education levels in the city's areas. Open statistics regarding education levels for parents with children under six were available for all area levels.

### 2.3 Data Analysis

The data were processed using Microsoft Excel, and statistical analysis was conducted using *R* in RStudio and IBM SPSS version 28.0.1.0. Parental education levels were categorised separately for mothers and fathers. To avoid random fluctuations between years, a 5‐year average from 2018 to 2022 was calculated for the explanatory variable ‘educational level’. As the number of children seeking burn injury treatment per year was relatively low, a 5‐year average was also calculated for this variable for the same reasons. Simple linear regression models were used to determine whether the explanatory variable of educational level in the area could explain the proportion of children receiving hospital care. The *R*‐squared value was of interest because it can capture how much of the variation parental education can explain per area. The data were analysed to determine statistical significance, with results considered significant at a *p* value of less than 0.05.

### 2.4 Ethical Considerations

No personal data were handled, as all available data were coded and reported at the area level, making it impossible to trace information back to individual persons [16].

## 3 Results

### 3.1 Descriptive Data

In Gothenburg, there was an average of 47,625 children aged 0–6 years between 2018 and 2022. Of these, 705 were treated for burn injuries at Queen Silvia Children's Hospital. This equates to an average of 141 children per year, or 0.3% of all children aged 0–6 years residing in Gothenburg.

The data were collected from 36 areas, with 5‐year averages calculated for pre‐upper secondary programme and post‐upper secondary programme education for both mothers and fathers. All descriptives are presented in Table 1.

**TABLE 1:** Column 1 lists areas, Column 2 the mean number of burns per area/year, Column 3 the mean number of children per area/year, Column 4 the mean proportion of children with burns per area/year and Columns 5–8 the mean proportion of parents by educational level.

			Parent, highest education level						
Area	Number of children in each area, 5‐year mean	Percentage of children (age 0–6) cared at hospital with burns, 5‐year mean (%)	Mothers, compulsory school (%)	Mothers, high school (%)	Mothers, college or higher (%)	Fathers, compulsory school (%)	Fathers, high school (%)	Fathers, college or higher (%)	Percentage of children (age 0–6) cared at hospital with injury, poisoning and certain other consequences of external causes, 5‐year mean (%)
Kortedala	1691	0.48	20	33	41	23	37	36	4.7
Frölunda Torg‐Tofta	779.4	0.44	16	31	47	20	36	41	4.1
Centrala Tynnered	1462.4	0.40	13	32	51	17	38	42	4.9
Bergsjön	2125.6	0.39	27	30	33	27	36	32	4.0
Östra Angered	679.4	0.38	4	31	62	8	44	47	5.2
Västra Biskopsgården	2320.4	0.38	26	30	36	24	36	35	4.3
Norra Angered	2009.6	0.38	19	37	36	26	41	28	4.1
Östra Biskopsgården	967	0.37	12	29	56	14	34	48	4.0
Södra Angered	2059.6	0.37	30	31	31	31	35	29	4.1
Kålltorp‐Torpa‐Björkekärr	1729	0.36	3	15	80	4	23	71	5.3
Guldheden‐Landala	949	0.36	3	14	82	4	18	76	4.3
Majorna‐Stigberget‐Masthugget	2198.4	0.35	3	11	86	3	20	75	4.1
Olskroken‐Redbergslid‐Bagaregården	826.6	0.34	3	17	79	4	28	67	4.8
Stora Högsbo	2283	0.33	4	22	72	6	30	62	4.3
Backa	2347	0.32	9	29	57	12	35	49	4.5
Lunden‐Härlanda‐Överås	1060.2	0.30	2	15	81	3	25	70	4.3
**Gothenburg total mean**	**47,625**	**0.30**	**10**	**25**	**63**	**11**	**32**	**55**	**4.3**
Bratthammar‐Önnered‐Näset	1101	0.29	1	16	81	2	27	70	4.8
Kyrkbyn‐Rambergsstaden	1315	0.29	4	21	72	7	28	62	4.4
Tuve‐Säve	1112.6	0.29	6	28	65	9	38	51	4.6
Centrala Angered	999	0.28	25	35	35	26	39	32	4.3
Södra Torslanda	1529.6	0.26	2	24	73	3	35	61	4.1
Kärra‐Rödbo	976.8	0.25	4	34	61	7	44	48	4.4
Kallebäck‐Skår‐Kärralund	911.8	0.24	2	15	81	3	23	72	4.4
Olivedal‐Haga‐Annedal‐Änggården	1273	0.24	1	12	84	2	18	78	3.6
Krokslätt‐Johanneberg	1129.2	0.23	3	16	76	4	21	72	3.9
Kvillebäcken	982.2	0.22	6	22	66	8	27	61	3.1
Norra Centrum	1317.2	0.20	2	19	74	4	26	66	4.3
Södra Skärgården	313	0.19	1	28	69	4	29	67	4.3
Askim‐Hovås	1255	0.19	4	18	76	4	27	67	4.4
Gamlestaden och Utby	1437.4	0.18	4	19	75	6	28	64	4.4
Björlanda	818.4	0.17	1	18	79	3	32	64	4.5
Kungsladugård‐Sanna	850.8	0.16	2	14	82	5	21	73	4.6
Norra Älvstranden	1043.2	0.15	1	15	82	4	21	73	3.8
Älvsborg	1586.6	0.15	0	12	87	2	18	79	4.3
Kärrdalen‐Slättadamm	828.2	0.14	4	19	75	6	27	65	4.8
Billdal	1358	0.09	1	15	83	3	21	74	3.9

### 3.2 Comparative Data

A relationship between parental education levels and children treated for burn injuries emerged, with significant results (*p* value < 0.001) identified regarding both fathers’ and mothers’ education levels. Children suffered burn injuries in all areas of Gothenburg, but where a higher proportion of parents had a maximum of pre‐upper secondary education, on average, a higher proportion of children were hospitalised for burn injuries (see Tables 1 and 2).

**TABLE 2:** Number of children aged 0–6 years who were treated for burns annually at Queen Silvia Children's Hospital.

Number of children that received care for burn injuries	2018	2019	2020	2021	2022	Total
	154	122	147	142	140	705

Conversely, in areas where a higher proportion of parents had a post‐upper secondary education or higher, a lower proportion of children were hospitalised for burn injuries. Similar trends emerged for both mothers and fathers, with the highest adjusted *R* ^2^ value occurring when the father had completed the post‐upper secondary programme (adjusted *R* ^2^ = 0.4) and the lowest when the mother had completed the pre‐upper secondary programme (adjusted *R* ^2^ = 0.38) (see Tables 3 and 4).

**TABLE 3:** The four regression models regarding children treated for burns and parents’ educational levels.

Dependent variable	Explanatory variable, proportions of parents with highest education level	Estimate (95% CI)	Sig^*^	Adjusted *R*‐squared
Percentage of children under the age of 6 cared at hospital for burns per area	Mothers, compulsory school	0.006 (0.003–0.01)	*p* < 0.001	0.35
	Mothers, college or higher	−0.003 (−0.005 to −0.002)	*p* < 0.001	0.37
	Fathers, compulsory school	0.007 (0.004–0.009)	*p* < 0.001	0.37
	Fathers, college or higher	−0.004 (−0.005 to −0.002)	*p* < 0.001	0.39

* Sig, significance *p* < 0.05 is statistically significant.

**TABLE 4:** Regression models explaining general injuries in relation to parental education.

Dependent variable	Explanatory variable, proportions of parents with highest education level	Estimate (95% CI)	Sig^*^	Adjusted *R*‐squared
Percentage of children aged 0–6 years who received hospital care for any injury, poisoning or other consequences of external causes	Mothers, compulsory school	−0.004 (‐0.021 ‐ 0.012)	*p* = 0.60	−0.02
	Mothers, college or higher	−0.001 (‐0.007 ‐ 0.009)	*p* = 0.80	−0.03
	Fathers, compulsory school	−0.004 (‐0.203 ‐ 0.013)	*p* = 0.66	−0.02
	Fathers, college or higher	−0.002 (‐0.011 ‐ 0.007)	*p* = 0.74	−0.03

* Sig, significance *p* < 0.05 is statistically significant.

This implies that almost 40% of the variation in children sustaining burn injuries could be explained by parental education levels.

Additional observations revealed differences in the proportion of parents with pre‐upper secondary and post‐upper secondary education levels across different areas. Among mothers, the highest level of college education was observed in Majorna–Stigberget–Masthugget, whereas the lowest level was observed in Södra Angered. For fathers, the lowest level of college education was found in Norra Angered, whereas the highest was observed in Guldheden (Table 1).

**FIGURE 1:**
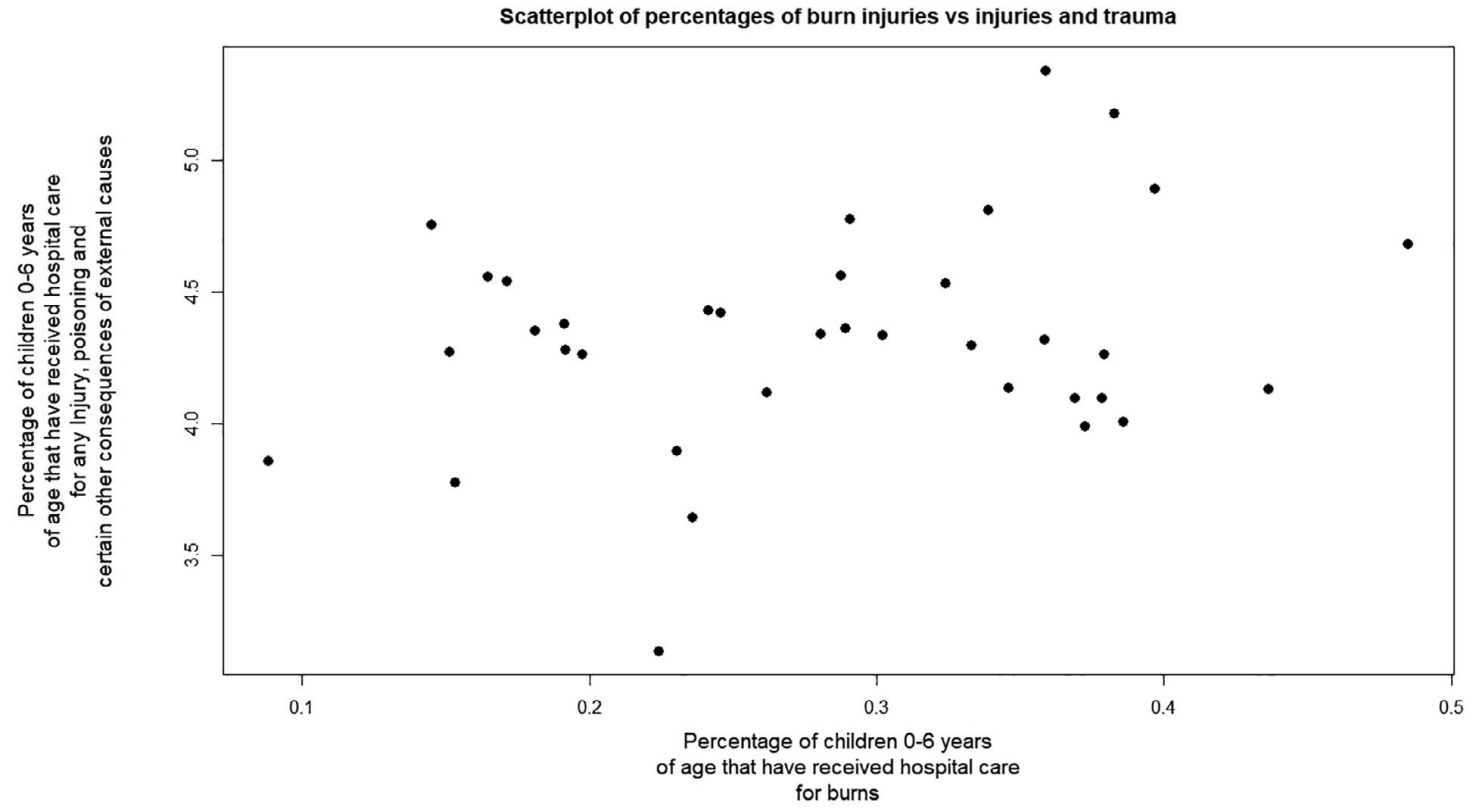
Comparison of the observed values of burn injuries (ICD codes T20–T25 and T29) with those of children who received hospital care for injury or trauma (ICD chapters S and T), with little to no association observed between these proportions.

To rule out the possibility that the proportion of children in areas with a higher incidence of burn injuries may also be exposed to a higher degree of other traumas and injuries, this study investigated the relationship between the proportion of burn injuries and diagnosed injuries and traumas. As seen in Figure 1, the proportion of children who were hospitalised with burn injuries and that of children with other traumas were not related. This linear regression model was found to be not significant (*p* = 0.12), with an *R*‐squared value of 0.07. The *R*‐squared value indicates a low association between the variables. The further evaluation of all educational proportions in this study was found to be not significant and had low *R*‐squared values (Table 4). Therefore, overall injuries and traumas did not act as a mediator.

When investigating the association between education level and the outcome of ICD chapters S and T, the adjusted *R* ^2^ values decreased substantially, showing no explanatory power and lacking statistical significance (Table 4).

## 4 Discussion

The results of this study demonstrate an association  between parental education level and young children's burn injuries, confirming that education can be a meaningful predictor of health [8]. Children from all areas of Gothenburg sustained burn injuries, regardless of their parents’ education levels; however, the areas with the lowest education levels recorded the highest burn injury rates. This relationship has been demonstrated in previous research [4, 5, 6]. The study's results indicate a connection between parents’ education levels and children's burn injuries, with education accounting for almost 40% of variation in such injuries. There were marginal differences depending on whether the mother's or father's education level was studied. Note that the father's education level showed the highest association, despite research often focusing on the mother [17]. In contrast, there was no relationship between parental education level and overall injury‐related hospital care (ICD chapters S and T). This might be due to multiple reasons, one being that the mechanism of injury varies considerably depending on the type of traumatic injury [18]. Another possibility is that additional sociodemographic factors not included in this study may act as confounders. However, further examining whether such factors significantly influence both educational level and the proportion of the population hospitalised for burn injuries is beyond the scope of this study.

Although this study does not explicitly identify the underlying causes of the observed relationship, the previous research has suggested parental knowledge of trauma as a contributing factor. For instance, in the context of dental injuries, parents often possess limited knowledge regarding appropriate responses. A significant proportion of paediatric dental injuries are presented to specialists after a delay, which reduces treatment effectiveness. This delay is likely attributable to a lack of parental awareness, ultimately leading to poorer clinical outcomes [19].

In addition, other studies have highlighted the critical role of environmental factors in the occurrence of unintentional injuries. These findings emphasise supporting parents in injury prevention and fostering safer home environments for children [20]. Child age also appears to be a significant factor. As infants gain mobility, parental supervision often decreases, whereas the risk and severity of injuries tend to increase [21].

This study supports the hypothesis that burn injuries can be linked to a certain education level, indicating inequality. This is consistent with the findings of Padalko et al. [6], who proposed various factors, such as lower income, living in rural settings and fewer years of parental education, to be linked to an increased risk for paediatric burn injuries. The presence of segregation and the differing frequency of burn injuries based on parental education level demonstrate that the Swedish Child Rights Act, which includes the United Nations Convention on the Rights of the Child as part of Swedish law [22], has not yet been fully implemented in society.

We argue that it is not the residential area itself that causes children's burn injuries. Area‐level patterns mirror the city's housing segregation, which affects socioeconomically vulnerable groups. It is crucial to distinguish between group‐ and individual‐level results. This study makes no claims about the individuals in these areas but identifies a group‐level trend.

The results suggest a persistent pattern of social vulnerability and injustice, underscoring the need for sustained sociopolitical intervention. Children's health and rights should not be contingent upon parental education or residential location. To safeguard children's right to health and care, it is imperative to allocate economic resources toward early interventions, prioritising areas and population considered at‐risk.

Burn injuries in children can often be prevented. Although relatively few children require hospital treatment for such injuries, the majority result from accidents. The Swedish child health service offers opportunities to create both general and targeted interventions with the potential to reach large population segments through paediatric specialist nurses [11]. This study shows that parents with a lower education level may be a group that should be given access to increased resources to prevent accidents, such as burns. This is an important follow‐up to the study. The proposed resources can be general, in the form of increased health literacy among healthcare providers, specifically through enhanced knowledge about risk groups and improved awareness of how information is conveyed. With increased knowledge, healthcare providers can better design person‐centred solutions that fully consider the child's context and provide support tailored to the family's needs and conditions. To create person‐centredness, individuals’ narratives must be part of the process [23]; therefore, parents’ perspectives and experiences should be investigated in future research. The goal of a person‐centred approach is to develop better interventions and increase health literacy. It is important to make information as accessible as possible to as many people as possible. Carlsson et al. [24] performed an intervention targeting families with lower education levels; the study provided extended information to improve risk precautions regarding burn injuries and scalds. It showed that preventive measures taken by the families improved after the intervention compared to the control group, who did not receive extended information [24].

According to the National Board of Social Affairs and Health and the Swedish child health units, all families with newborns at 8 months of age should be offered a home visit. Despite this, the variation in accessibility of home visits is remarkably high across different areas and families [25]. The socioeconomic situation plays a crucial role, and it is unacceptable that families’ circumstances vary depending on their residential areas rather than their actual needs, especially when most accidents have been observed to occur at home [26, 27]. A total of 96% of all scalding injuries occur in the home environment [26]. Kendrick et al. [27] showed that safety information was most effective when delivered in the family home. The study suggested that preventive measures in the form of information can reduce the risk of accidents, particularly if the conversation takes place in the home through home visits.

This study contributes to increased knowledge, and its findings could be used to inform target populations for intervention and education programmes. It also highlights the need for further research that includes families’ experiences of the information provided for health promotion and accident prevention purposes. Additionally, future studies need to evaluate the effects of targeted interventions, which could advantageously include a participatory design.

### 4.1 Strengths and Weaknesses

This study included only children requiring hospital treatment. Consequently, the results cannot address the total number of burn injuries, as the study did not examine families managing burns through home care or seeking treatment at primary care centres. However, this selection likely represents the most severe burn injuries treated at the hospital, which can also be considered a strength. The association effectively highlights the relationship between more severe injuries and parental education levels, as the results are not confounded by the inclusion of minor injuries.

The small number of children participating may affect the transferability and generalisability of the results. Another potential source of error was that data collection was undertaken during the COVID‐19 pandemic. When examining data from these years, no significant differences or clear patterns were observed; therefore, these data were not handled separately.

Furthermore, this study examined effects at the area level, which does not allow for statements at the individual level. The study differs from the previous research because it focuses solely on education levels without considering other health factors or confounders, such as overcrowding, economic conditions or other health statuses.

Investigating the impact of education level is relevant and important, but education represents only one factor with a relatively high explanatory power. A study that accounts for confounders might provide more robust results in some respects.

## 5 Conclusion

An association was found between parental education levels and the incidence of burn injuries in children. However, this association was not observed for general injuries. These findings pertain to Gothenburg and are limited by the scope of the available data. Future studies could explore the relationship between different types of burn injuries and the educational level of parents. In addition, research is needed at the individual level, along with studies that consider the perspectives of families.

## Author Contributions

**Gabriela Valladares Solano:** conceptualization, manuscript acquisition, methodology, writing review and editing. **Emma Kos:** conceptualization, manuscript acquisition, methodology, writing review and editing. **Mattias Wennergren:** conceptualization, manuscript acquisition, methodology, formal analysis, writing review and editing. **Ann‐Sofie Frisk Cavefors:** conceptualization, manuscript acquisition, methodology, writing review and editing. **Johanna Räntfors:** conceptualization, manuscript acquisition, methodology, writing review and editing. **Stefan Nilsson:** conceptualization, manuscript acquisition, methodology, writing review and editing.

## Ethics Statement

The study received ethical approval from the Swedish Ethical Review Authority (2023‐05824‐0).

## Conflicts of Interest

The authors declare no conflicts of interest.

## Data Availability

The raw data supporting the conclusions of this article will be made available by the authors upon reasonable request.
